# The challenging diagnosis of sinistral portal hypertension in a patient with liver cirrhosis: a case report and literature review

**DOI:** 10.3389/fgstr.2026.1729501

**Published:** 2026-04-20

**Authors:** Natalia Altarsha, Adrian Raoul Kobe, Emanuel Burri, Magdalena Filipowicz Sinnreich

**Affiliations:** 1 Department of Gastroenterology and Hepatology, University Department of Medicine, Cantonal Hospital Baselland, Liestal, Switzerland; 2 Department of Radiology and Nuclear Medicine, University Hospital Basel and University of Basel, Basel, Switzerland; 3 Department of Biomedicine, Liver Immunology, University Hospital Basel and University of Basel, Basel, Switzerland

**Keywords:** ascites, cirrhosis, left-sided portal hypertension (LSPH), percutaneous transluminal angioplasty (PTA), portal hypertensive gastropathy, splenic vein stenosis

## Abstract

**Background:**

Left-sided portal hypertension (LSPH) is commonly associated with pancreatic disease and typically manifests with gastrointestinal bleeding, but not with ascites or hypoalbuminemia. The coexistence of LSPH with liver cirrhosis complicates diagnosis and management, as standard imaging often fails to identify the underlying cause of portal hypertension-related complications.

**Case presentation:**

We report a case of a 59-year-old man with decompensated alcohol-related cirrhosis and prior pancreatic surgery, who presented with recurrent gastrointestinal bleeding, severe hypoalbuminemia, and treatment-refractory ascites, all of which persisted despite placement of a transjugular intrahepatic portosystemic shunt (TIPS). Diagnostic work-up included repeated imaging and hemodynamic measurements. Importantly, invasive splenoportography was the only approach that allowed the detection of a hemodynamically significant splenic vein stenosis, and subsequent percutaneous transluminal angioplasty (PTA) led to rapid clinical improvement.

**Key finding:**

Standard imaging failed to detect a splenic vein stenosis, which was identified only by invasive phlebography performed via the already placed TIPS. Following PTA, ascites resolved, serum albumin normalized, and no further gastrointestinal bleeding occurred. To our knowledge, this is the first case report describing the full reversal of such a constellation of symptoms after treatment of splenic vein stenosis.

**Conclusion:**

This case demonstrates that splenic vein stenosis can cause both hemorrhagic and non-hemorrhagic complications in patients with cirrhosis. Recognition and interventional treatment of LSPH may reverse refractory ascites and hypoalbuminemia, highlighting the importance of high clinical suspicion and invasive evaluation in complex portal hypertension cases.

## 1 Introduction

Left-sided portal hypertension (LSPH) is typically associated with pancreatic disease and usually causes upper gastrointestinal bleeding, but not ascites or hypoalbuminemia (1–8). The coexistence of LSPH and liver cirrhosis complicates diagnosis and management (9–11). Imaging and hemodynamic studies are important for differentiating LSPH from generalized portal hypertension (3, 4, 10–13). We report the case of a 59-year-old man with liver cirrhosis and a history of pancreatic surgery who, despite a functional transjugular intrahepatic portosystemic shunt (TIPS), presented with recurrent gastrointestinal bleeding, treatment-refractory ascites, and severe hypoalbuminemia. Standard imaging failed to identify a cause. Invasive splenoportography revealed a high-grade stenosis of the splenic vein at its confluence with the portal vein. Following percutaneous transluminal angioplasty (PTA), ascites rapidly resolved, albumin levels normalized, hemoglobin stabilized, and recurrent bleeding did not occur. This case demonstrates that splenic vein stenosis can cause both hemorrhagic and non-hemorrhagic complications in cirrhotic patients, and that recognition and treatment of LSPH can reverse treatment-refractory ascites and hypoalbuminemia.

## 2 Case presentation

A 59-year-old man with known decompensated liver cirrhosis due to alcohol-related liver disease (ALD) presented in August 2024 with recurrent episodes of decompensation, including gastrointestinal bleeding and ascites, despite having undergone placement of a TIPS four months earlier in April 2024 for refractory ascites, which had required regular paracenteses since January 2024. A gastroscopy in May 2024 had revealed small esophageal varices without risk stigmata, in addition to portal hypertensive gastropathy. Carvedilol treatment was not tolerated because of hypotension and deterioration of renal function.

Laboratory findings in August 2024 revealed treatment-refractory hypoalbuminemia (albumin 24 g/L) despite a protein-rich diet and regular albumin supplementation administered during large-volume paracenteses, which were performed regularly despite a patent TIPS. Diuretic treatment was challenging because of hypotension and frequent deterioration of renal function. The patient had impaired liver function (Child-Pugh class B, 9 points, and a MELD score of 10 points). In addition, there was bicytopenia with platelets at 130×10^9^/L and iron-deficiency anemia (hemoglobin 90 g/L) despite regular iron supplementation. As the TIPS remained patent, there were no further options for therapeutic escalation, and the patient was referred to a transplant center for discussion of potential listing.

**Figure 1:**
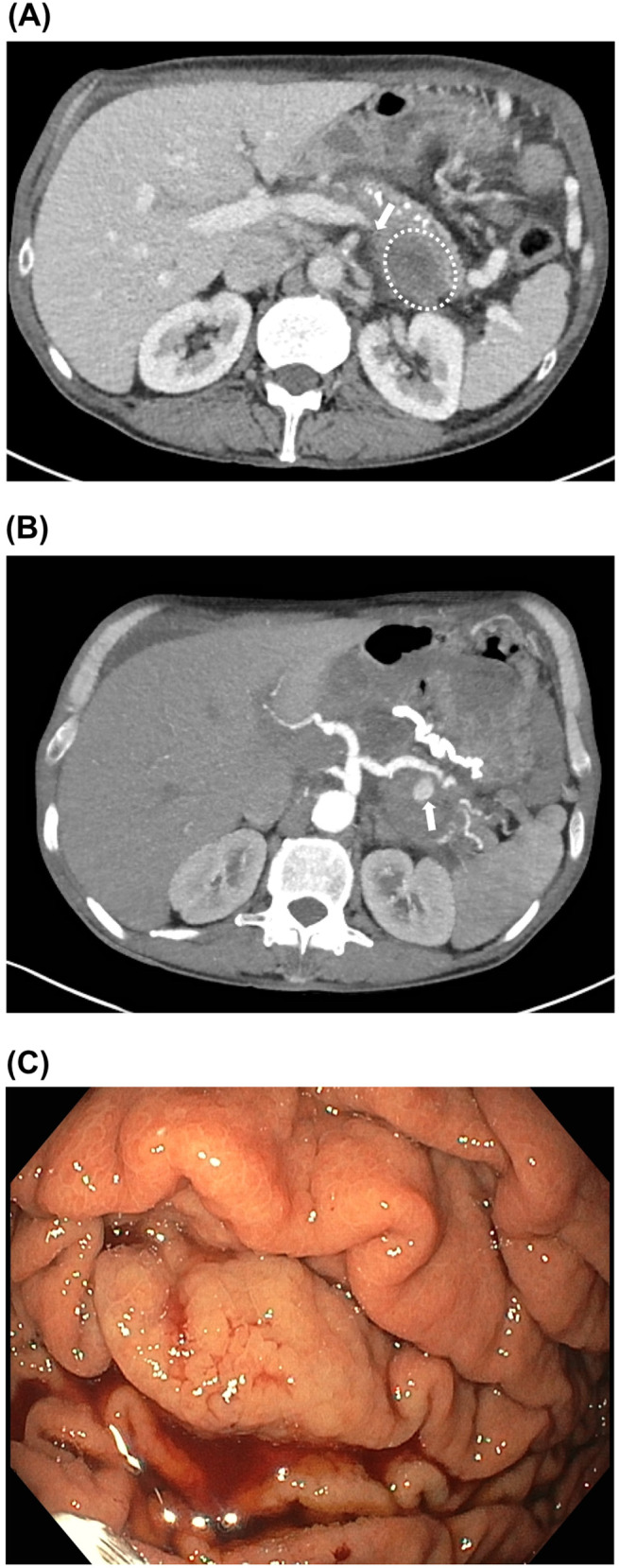
Pancreatitis with complications and portal hypertensive gastropathy. **(A)** CT scan performed in July 2017 showing severe flare of chronic pancreatitis with presumed thrombosis/compression of the *V. lienalis* (white arrow) and a walled-off necrosis (dotted circle). **(B)** CT scan from July 2017 showing the pseudoaneurysm of the *V. lienalis* as a complication of the severe pancreatitis. **(C)** Gastroscopy in August 2024 showing signs of portal hypertensive gastropathy with minimal fresh bleeding.

The patient had a history of chronic alcohol-induced pancreatitis, requiring substitution of pancreatic enzymes, with three flares between December 2015 and October 2016, in addition to complex, extensive pancreatic pseudocysts, and severe chronic pain. A severe episode of pancreatitis in June 2017 was accompanied by complications including a walled-off necrosis (WON), gastric variceal bleeding in July 2017 (treated with histoacrylate injection), due to suspected splenic vein thrombosis (although substantial splenic vein compression due to pancreatitis with large pseudocysts and WON cannot be excluded; **Figure 1A**), and subsequent splenic artery coiling, the latter performed due to a splenic artery aneurysm and arterial bleeding into a pseudocyst (**Figure 1B**). In the further course, the patient also underwent a duodenum-preserving pancreatic head resection, pancreatic duct incision, and a latero-lateral pancreaticojejunostomy (Roux-en-Y reconstruction), which was electively performed in July 2018 due to recurrent severe pancreatitis with chronic pain and complex pseudocysts. In subsequent imaging, namely, computed tomography (CT) scans dating from October 2019, there was no evidence of splenic vein thrombosis despite the patient not having been treated with anticoagulation.

A transjugular liver biopsy with Hepatic venous pressure gradient (HVPG) measurement in February 2022 revealed an HVPG of 8 mmHg and histology compatible with cirrhosis due to ALD. Steatosis was absent, compatible with abstinence from alcohol since 2017. In the portal tracts and septa, a mild to moderate, partly mixed inflammatory infiltrate was observed.

In late August 2024, the patient suffered a massive upper gastrointestinal bleed with hematemesis and melena. Esophagogastroduodenoscopy revealed severe portal hypertensive gastropathy with active bleeding, which was treated with adrenaline injection and hemostatic powder (**Figure 1C**). Small esophageal varices without signs of bleeding and no fundal varices were noted. Argon plasma coagulation (APC) treatment was not performed because of the generalized vulnerability of the gastric mucosa.

Imaging with CT (April, September, and October 2024) and repeated Doppler ultrasound (August – October 2024) demonstrated extensive four-quadrant ascites, no focal liver lesions, a patent TIPS in proper position with adequate flow velocities, and no evidence of portal vein or splenic vein thrombosis.

After another massive portal hypertensive bleed, an invasive TIPS assessment was performed in October 2024, during which a stenosis at the junction of the stent and the right hepatic vein was discovered. PTA dilation and proximal extension with a stent graft were performed, reducing the HVPG from 17 to 7 mmHg. Such stenosis typically occurs when the TIPS stent is slightly too short relative to the length of the parenchymal tract of the hepatic vein. This can lead to turbulent flow and focal intimal hyperplasia at the junction with the inferior vena cava (14). Accordingly, the stent was extended cranially into the right hepatic vein, resulting in a sustained hemodynamic improvement.

**Figure 2:**
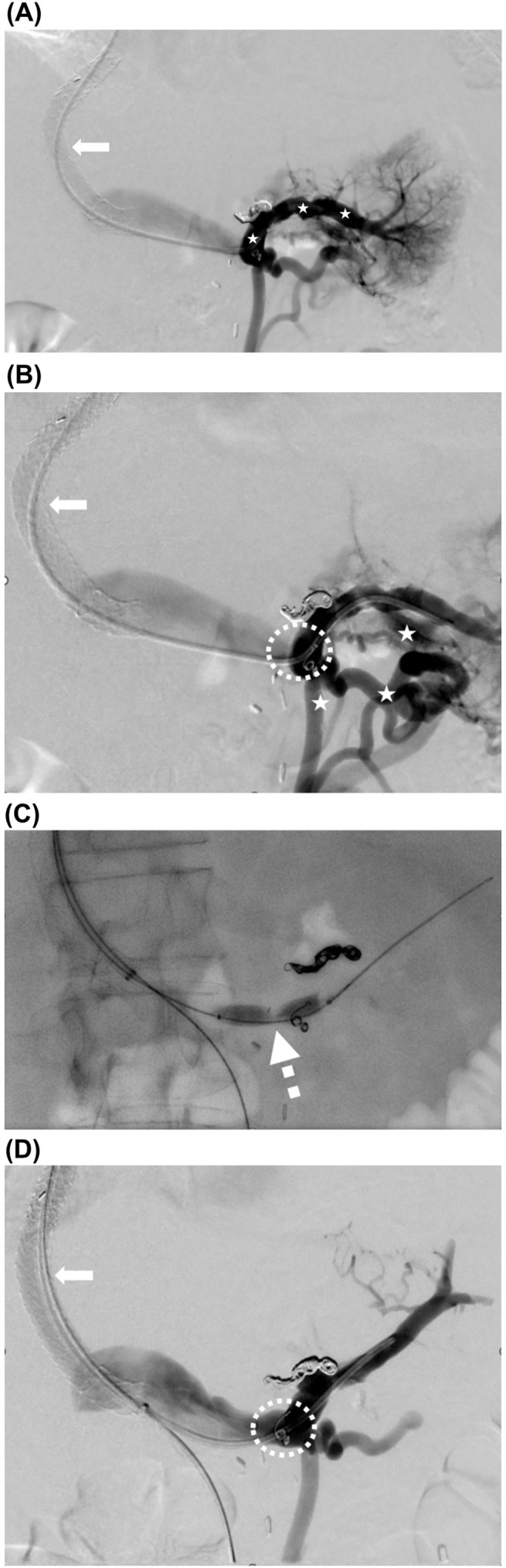
Percutaneous transluminal angioplasty of a high-grade splenic vein stenosis. Technically successful PTA of a high-grade splenic vein stenosis at its confluence with the portal vein. **(A, B)**. Transjugular splenoportography via a catheter in the splenic vein (placed through the TIPS, white arrow) in November 2024. **(A)** shows no evidence of splenic vein thrombosis (white stars), and **(B)** shows numerous variceal veins (white stars) with reduced flow in the portal vein due to a significant focal stenosis of the splenic vein (dotted circle). The pressure gradient between the splenic and portal veins was 7 mmHg. **(C)** Percutaneous transluminal angioplasty (12 mm PTA) in December 2024 demonstrating demarcation of the focal stenosis (dotted arrow). **(D)** Final splenoportography showing resolution of the stenosis (dotted circle) and improved outflow through the TIPS (white arrow). The final pressure gradient was reduced to 1 mmHg.

Despite this intervention, the patient continued to experience recurrent gastrointestinal bleeding and refractory ascites. In parallel, the work-up for liver transplantation proceeded. CT imaging in October and November 2024 demonstrated the persistent presence of perisplenic and gastric varices. Because no stenosis of the splenic vein could be visualized — even retrospectively on CT scans from April, September, and October 2024 — an invasive assessment with splenoportography and pressure measurements was performed in November 2024. The shunt was patent with normal flow and an HVPG of 8 mmHg; the splenic vein showed no evidence of thrombosis (**Figure 2A**). However, a severe, hemodynamically significant stenosis, or membranous occlusion, of the splenic vein at its confluence with the portal vein was identified (**Figure 2B**), which was presumed to be responsible for the persistent severe congestive gastropathy.

In the further course, the patient developed septic shock in November 2024, presumably due to aspiration pneumonia, and a non-ST-segment elevation myocardial infarction (NSTEMI) in the context of two-vessel coronary artery disease. Percutaneous transluminal coronary angioplasty was performed. During the same hospital stay (December 2024), after interdisciplinary discussion, a PTA (12mm) of the severe splenic vein stenosis due to a membranous web at its confluence into the portal vein was performed, reducing the pressure gradient between the splenic vein and portal vein from 7 to 1 mmHg (**Table 1**, **Figures 2C, D**).

**Table 1:** Pressure measurements of the corresponding radiology report in December 2024.

Manometry	Before PTA (mmHg)	After PTA (mmHg)
Central venous pressure	11	10
Direct portal vein pressure	14	18
Splenic vein pressure	21	19

Reduction of the pressure gradient between the splenic vein and the portal vein from 7 mmHg to 1 mmHg. The HVPG increased from 3 mmHg to 8 mmHg after PTA of the splenic vein.

Over the next two weeks, the patient experienced marked and rapid clinical improvement, with normalization of albumin and stabilization of hemoglobin at 100 g/L (**Table 2**). Ultrasound showed only minimal ascites without the need for paracentesis. International normalised ratio (INR) remained stable at 1.4, and renal function improved after reducing diuretic therapy. The Child-Pugh score improved to 5 (Class A). Notably, normalization of albumin after splenic vein PTA and consequent resolution of ascites suggest that prior protein loss and recurrent bleeding were due to severe portal hypertensive gastropathy caused by local sinistral portal hypertension.

**Table 2:** Laboratory data.

Variable	Reference range	11.2024	2 weeks after PTA	6 months after PTA
Complete blood count				
White-cell count (10^9^/l)	3.9-10.2	7.6	5.4	6.1
Hemoglobin (g/L)	135-172	69	100	130
Platelets (10^9^/l)	150-370	248	225	144
Routine chemistry				
Sodium (mmol/L)	136-145	131	130	134
Potassium (mmol/L)	3.5-5.1	4.0	4.6	5.0
BUN (mmol/L)	2.7-6.8	4.9	6.5	5.9
Creatinine (mg/dl)	0.67-1.18	0.78	0.84	1.15
Hepatic function panel				
Albumin (g/l)	35-50	22	37	40
Total bilirubin (mg/dl)	<1.17	0.44	0.36	1.59
ALP (U/L)	40-130	63	78	137
AST (U/L)	<41	14	38	100
ALT (U/L)	<41	<5	<5	16
Coagulation studies				
INR	<1.2	1.4	1.4	1.3

AST, aspartate aminotransferase; ALT, alanine aminotransferase; ALP, alkaline phosphatase; BUN, Blood urea; INR, international normalized ratio.

A gastroscopy was performed three months later, in March 2025, due to suspected upper gastrointestinal bleeding. It showed grade D reflux esophagitis with an ulcer, with no evidence of varices and no significant portal hypertensive gastropathy. Laboratory testing in July 2025 demonstrated persistently normal albumin levels, and ultrasound revealed only minimal ascites, indicating a sustained response following the PTA intervention of the splenic vein (**Table 2**).

## 3 Discussion

Most published reports of LSPH describe patients with isolated pancreatic disease, such as pancreatitis, pancreatic neoplasms, or post-pancreatectomy states, without underlying cirrhosis (1, 2, 5, 6, 15–20). The pathophysiology of LSPH is distinct: splenic vein obstruction results in segmental venous hypertension, leading to gastric and, less commonly, colonic varices with a significant risk of gastrointestinal bleeding (3, 6, 13, 20, 21). In contrast, portal hypertension in cirrhosis is caused by increased intrahepatic resistance and splanchnic vasodilation, resulting in the development of esophageal and gastric varices, portal-hypertensive gastropathy, ascites, and is frequently accompanied by impaired hepatic synthetic function, including hypoalbuminemia and hyperbilirubinemia (9–12, 14, 22, 23).

What makes our case exceptional is that the patient not only developed recurrent gastrointestinal bleeding — most likely related to portal hypertensive gastropathy due to LSPH — but also exhibited refractory ascites and hypoalbuminemia, which are classically considered complications of cirrhosis. Remarkably, these latter complications did not improve after TIPS placement but resolved rapidly following angioplasty of the splenic vein stenosis. This strongly indicates that both the patient’s bleeding episodes and the non-hemorrhagic manifestations were primarily driven by LSPH rather than cirrhosis. To our knowledge, no previous case reports have described such a presentation, in which recurrent bleeding, ascites, and hypoalbuminemia were fully reversible after treatment of splenic vein stenosis (1, 2, 4–6, 8, 13, 15–20, 24–27). We assume that the recurrent bleeding episodes and recurrent ascites after TIPS placement were caused by the splenic vein stenosis, which had not been identified on repeated imaging. Because a TIPS dysfunction had been found with invasive imaging and hemodynamic measurements in October 2024 (which was previously not identified on CT imaging), an additional LSPH had not been initially suspected and thus had not been invasively investigated.

The available literature consistently emphasizes that ascites is not a typical feature of isolated LSPH and thus is considered a sign of generalized portal hypertension due to cirrhosis (5, 6, 9–11, 14). The majority of published case reports of LSPH, including those related to pancreatitis, pancreatic tumors, vascular compression, or splenic torsion, describe patients with normal liver function and no concomitant cirrhosis (1, 13, 15–17, 20). Even in rare descriptions of coexisting cirrhosis and LSPH, ascites and protein loss have not been directly attributed to splenic vein stenosis, nor shown to resolve after its treatment (1, 4, 13, 16, 18, 19, 28). This clearly distinguishes our patient from all previously reported cases. We suspect severe gastric protein loss via severe portal hypertensive gastropathy, given the rapid and durable improvement of hypoalbuminemia after PTA of the splenic vein stenosis.

Gastrointestinal bleeding remains the predominant complication of LSPH. Gastric varices, particularly cardiofundal varices (IGV1, GOV2), bleed in 16–45% of patients over 3 years, with higher mortality and rebleeding rates compared to esophageal varices (11). Gastric varices represent 17–25% of variceal manifestations in patients with cirrhosis (11). Portal hypertensive gastropathy (PHG) is also frequent in cirrhosis, though it more commonly causes chronic rather than acute bleeding (10, 11, 28). In our case, the patient indeed suffered from recurrent severe gastrointestinal bleeding, in addition to refractory ascites and hypoalbuminemia, an unusual constellation in this clinical setting. The patient had also suffered a gastric variceal bleeding in 2017, at that time caused by a splenic vein thrombosis that may have preceded the stenosis diagnosed only in 2024.

Our case highlights the diagnostic challenges that are associated with LSPH, especially in the context of concomitant chronic liver disease. Splenic vein stenosis is often missed on abdominal CT, with up to 22% of cases overlooked, especially in post-surgical or post-inflammatory settings (6, 20). In our patient, the diagnosis was only established by direct invasive visualization of the splenic vein via the previously placed TIPS. This underscores the need for high clinical suspicion and angiographic evaluation when cross-sectional imaging is inconclusive. This is particularly relevant in patients with a history of alcohol abuse, as the coexistence of ALD and pancreatitis occurs in approximately 15–25% of affected patients (29–31).

Therapeutically, PTA and stenting of the splenic vein are increasingly recognized as effective for LSPH-related bleeding (8, 11, 15, 16, 24–26). Comparative studies suggest lower rebleeding rates after stenting compared with splenic artery embolization (SAE) (4, 7, 8, 13, 18, 19, 26). SAE reduces LSPH by decreasing arterial inflow to the spleen, which in turn lowers splenic venous outflow and pressure within the splenic vein and its collateral pathways. This mechanism directly reduces the pressure driving variceal formation and bleeding in LSPH (7, 8, 32, 33). The effect is most pronounced in patients with large spleen volume or a high spleen/liver volume ratio.

No comparative studies are available directly comparing stenting versus PTA for the treatment of LSPH-related bleeding. The American Association for the Study of Liver Diseases notes that splenic vein stenting and SAE are both considered for LSPH, but does not address PTA as a primary modality for LSPH-related bleeding (11). Given that our patient was being considered for liver transplantation, we opted to avoid stenting the stenosis as an initial approach.

Our case expands this therapeutic spectrum by demonstrating that PTA of the splenic vein can not only control recurrent bleeding, but also reverse non-hemorrhagic complications, namely, refractory ascites and hypoalbuminemia, when these are driven by LSPH.

## 4 Conclusion

This case highlights an unusual constellation of clinical features in LSPH: refractory ascites with severe hypoalbuminemia, combined with severe portal-hypertensive gastropathy. These manifestations may represent a novel clinical expression of LSPH, possibly influenced by the coexistence of advanced liver disease/cirrhosis. Furthermore, our case underscores important diagnostic considerations: in patients with portal hypertension, recurrent bleeding, and ascites unresponsive to conventional therapies, splenic vein stenosis should be considered even when cross-sectional imaging does not demonstrate such a finding. In such settings, particularly in patients with a history of pancreatic disease, invasive splenoportography may be essential to establish the diagnosis. This case illustrates the broad clinical spectrum of LSPH and emphasizes the diagnostic challenges of splenic vein stenosis and the therapeutic relevance of its recanalization in cirrhotic patients.

## Data Availability

The original contributions presented in the study are included in the article/supplementary material. Further inquiries can be directed to the corresponding author.
